# Modeling Liquids and Resin-Based Dental Composite Materials—A Scoping Review

**DOI:** 10.3390/ma15113759

**Published:** 2022-05-24

**Authors:** Gaetano Paolone, Claudia Mazzitelli, Uros Josic, Nicola Scotti, Enrico Gherlone, Giuseppe Cantatore, Lorenzo Breschi

**Affiliations:** 1 Dental School, IRCCS San Raffaele Hospital, Vita-Salute University, 20132 Milan, Italy; gherlone.enrico@hsr.it (E.G.); cantatore.giuseppe@hsr.it (G.C.); 2 Department of Biomedical and Neuromotor Sciences (DIBINEM), Alma Mater University of Bologna, Via S. Vitale 59, 40125 Bologna, Italy; claudia.mazzitelli@unibo.it (C.M.); uros.josic2@unibo.it (U.J.); lorenzo.breschi@unibo.it (L.B.); 3 Department of Surgical Sciences, Dental School Lingotto, University of Turin, Via Nizza 230, 10126 Turin, Italy; nicola.scotti@unito.it

**Keywords:** resin-based composites, modeling resins, bonding agents, lubricants, wetting agents

## Abstract

Several lubricant materials can be used to model resin-based composites (RBCs) during restorative procedures. Clinically, instruments or brushes are wet with bonding agents (BAs) or modeling liquids (MLs) for sculpturing purposes. However, a knowledge gap exists on their effects on the mechanical properties of RBCs, requiring greater insight. Five databases were searched, including 295 in vitro studies on the use of lubricant materials for modeling RBCs during restorative procedures. Only articles in the English language were included, with no limits on the publication date. The last piece of research was dated 24 March 2022. In total, 16 studies were included in the review process, together with a paper retrieved after screening references. A total of 17 BAs and 7 MLs were investigated. Tensile (*n* = 5), flexural strength (*n* = 2), water sorption (*n* = 2), color stability (*n* = 8) and translucency (*n* = 3), micro-hardness (*n* = 4), roughness (*n* = 3), degree of conversion (*n* = 3), and monomer elution (*n* = 2) tests were carried out. In general, a maximum of 24 h of artificial storage was performed (*n* = 13), while four papers tested the specimens immediately. The present review identifies the possibilities and limitations of modeling lubricants used during restorative procedures on the mechanical, surface, and optical properties of RBCs. Clinicians should be aware that sculpturing RBCs with modeling resins might influence the composite surface properties in a way that is material-dependent.

## 1. Introduction

Restorative procedures, performed for therapeutic or esthetic purposes, fall with daily clinical practice, representing the most-performed dental treatment even in the early period of the COVID-19 pandemic when demand for the remaining dental services was substantially reduced [1]. Resin-based composites (RBCs) represent the material of choice for direct restorative procedures [2,3,4]. Adhesive dentistry has revolutionized restorative practice. Dentists have at their disposal a myriad of bonding systems to increase the retention and durability of composite restorations. Bonding systems have been categorized according to the clinical steps necessary to obtain the hybridization of dental tissues [5]. Over time, there has been an attempt to reduce the number of clinical steps and simplify adhesive procedures. Hence, there has been a shift from etch-and-rinse (ER) systems (2- or 3-step systems) to self-etch (SE) systems (2- or 1-step systems) to the latest system in the market—the universal adhesive (UA) system. The latter has been enthusiastically received by clinicians because of their clinical versatility, being able to be applied in both etch-and-rinse, self-etch, and selective enamel etching modes, as well as possibilities for application to both dental tissues and different restorative materials in a simple and time-saving manner [4]. While the simplification of adhesive systems has brought unquestionable operational advantages, the chemical complexity they refer to in order to incorporate into a single product all the components necessary for conditioning and adhesion can create incompatibility phenomena and result in a deficit in restoration durability [6,7].

The improvements in the chemistry of bonding systems have allowed the use of RBCs even in large cavities, with or without cuspal involvement [8]. In these cases, however, the procedural technique involves the sequential layering of the composite material with the possibility of shaping the material to achieve the original anatomy of the tooth. Esthetic and functional restorations are achieved through mimetic reproduction of natural dental anatomy, which is, in turn, realized through the comprehension of dental morphology, composite modeling techniques, and composite characteristics (color, shade, consistency, etc.) [9]. Depending on their chemical composition, resin composites are available in different stickiness, veering from filamentous to more compacted structured materials. From a clinical point of view, more sticky composites are difficult to handle and sculpt, requiring use by an expert hand. In order to enhance resin composite handling, reduced surface tension, increased wettability during anatomical tooth modeling, and modeling lubricating materials have been proposed to be used with brushes or moistened instruments [10].

Alcohol as a modeling material to prevent the sticking of the resin composite has been used for over 40 years. Although the alcohol does not influence the adaptation of the restorative material to the cavity wall [11], its use can damage the resin matrix and detrimentally worsen the mechanical properties of RBCs [12]. Therefore, clinical recommendations have been modulated over time.

From a clinical perspective, it is not uncommon for dentists to use bonding agents (BAs), which are routinely used in adhesive procedures as modeling lubricants to reduce the stickiness of the RBCs and enhance their handling capability [13]. This approach has aroused concerns about possible adverse effects on RBC characteristics, independent of whether the BA is an ER or SE, and the situation has been demonstrated to be more negative when dealing with UAs [5]. Indeed, the presence of hydrophilic monomers and solvents negatively affects the mechanical properties and surface stability of RBCs with respect to non-solvated and less hydrophilic BAs. The high solvent content (i.e., alcohol, acetone) is responsible for scarce polymerization phenomena, resulting in diminished bond reliability [14].

Some manufacturers have introduced peculiar materials for enhancing RBC materials’ handling during restorative procedures, generally referred to as modeling liquids (MLs), modeling resins, modeling agents, composite lubricants, and composite primers. These lubricants are used to wet dental composite instruments (i.e., spatula, plugger, condenser, probe) and brushes for every applied composite increment (between layers) and/or on the final layer to produce a smooth and esthetically delightful surface. The latter procedure is generally performed to save valuable time during finishing and polishing procedures [13,15]. Basically, MLs are unfilled resins comprised of methacrylates such as urethane dimethacrylate (UDMA), bisphenol A-glycidyl methacrylate (Bis-GMA), and triethylene glycol dimethacrylate (TEGDMA). MLs are mainly composed of hydrophobic non-solvated resins with scarce or no organic fillers, eventually not influencing the composite’s structure [16]. Even though MLs share the same methacrylate derivatives with the organic matrix of RBCs, chemo-mechanical alterations have been observed in the composite restorative materials after being modeled with the instrument lubricated with the ML. Indeed, the incorporation of compounds into the sculptured composite causes structural disruption [17], leading to many manufacturers not recommending the combined use of these resinous blends.

The effects of lubricants on surface hardness and the mechanical and optical properties of RBCs have been previously investigated. Scoping reviews are carried out to identify knowledge gaps by scoping a body of literature, clarifying concepts, and investigating research conduct. Although they are not conducted for the same purposes as systematic reviews, they still require transparent methods in their conduct in order to ensure that the results are valid [18]. Notwithstanding modeling liquids are not new to the market, data on their effects on composite properties in the literature are scarce and conflicting. Therefore, this scoping review aims to offer a comprehensive overview of the literature on the effect of different lubricants used in combination with RBC materials.

## 2. Materials and Methods

The present study followed the Preferred Reporting Items for Systematic Reviews and Meta-Analyses (PRISMA) extension for scoping reviews in terms of: (1) identification of the research question, (2) identification of relevant studies (keywords and databases), (3) determination of inclusion and exclusion criteria, (4) data extraction, and (5) summary of the results.

### 2.1. Identification of the Research Question

This review aims to analyze studies published in the field of dental biomaterials and restorative dentistry that have examined the use of different lubricants (namely, as bonding agents or modeling liquids) in combination with RBCs and their effects on composite surface characteristics.

### 2.2. Identification of Relevant Work

Two main domains were identified: “resin-based composites” and “lubricants”. Accordingly, a derivative sequence of keywords and free terms was developed (Table 1).

To ascertain potentially relevant studies, an electronic search of the literature was conducted on five databases (Cochrane, Embase, Medline, Scopus, Web of Science) with no limit on the publication date. The last search was carried out on 4 February 2022. The search strategy was defined by a professional librarian (I.M.) and further refined through team discussions (Table 2).

### 2.3. Determination of Inclusion and Exclusion Criteria

The inclusion criteria were in vitro studies in the English language published in peer-reviewed journals with no limits to the publication date. The exclusion criteria were: (1) papers not including BAs or MLs as modeling lubricants; (2) papers using BAs or MLs after finishing and polishing procedures only to provide a shiny effect to the restorations and not focusing on the effects on the surface characteristics of RBCs; (3) studies not written in English.

### 2.4. Data Extraction

**Figure 1:**
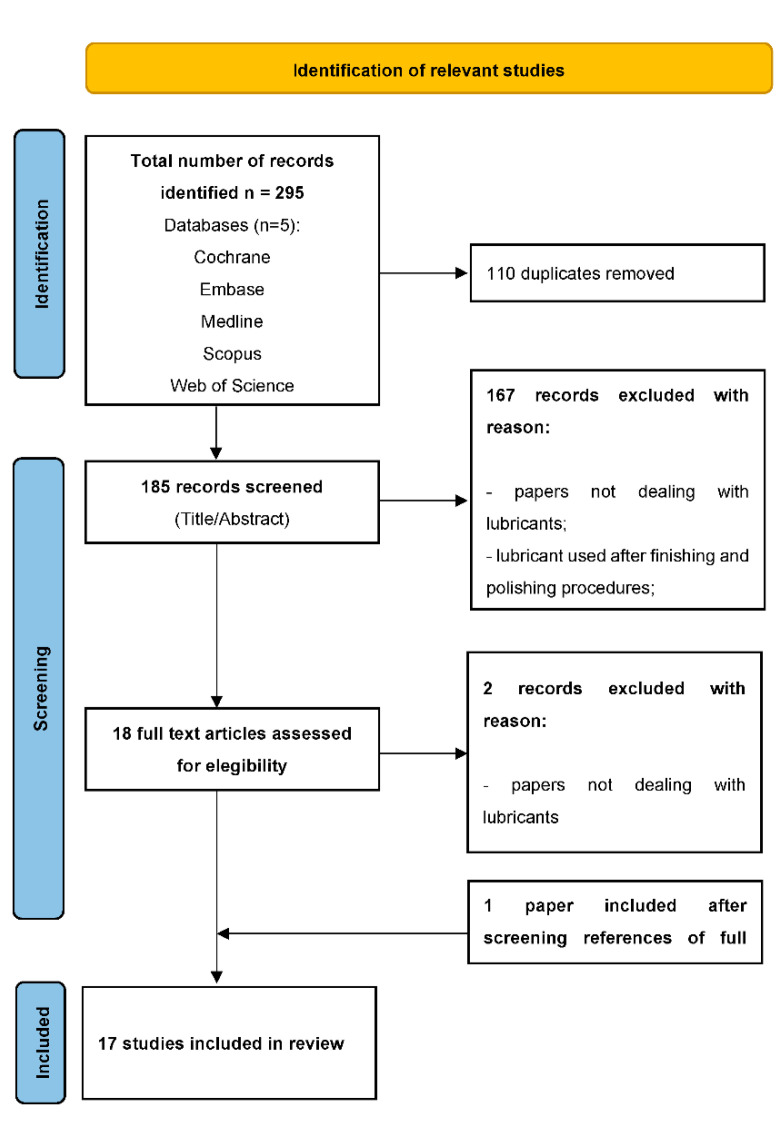
Flow chart showing the process followed for the identification of relevant studies. The first title and abstract screening obtained 295 records. After the revision and identification of the eligibility criteria, a total of 17 in vitro studies were included in the scoping review.

To ensure the consistency of the reviewing process, first, all authors screened the first 50 publications, which were randomly selected, discussed the results, and extracted data manually. Then, two independent reviewers (G.P. and C.M.) sequentially examined the titles and abstracts. Afterward, three reviewers (G.P., C.M., and N.S.) evaluated the full texts for potentially relevant studies. Disagreements on study selection were resolved by consensus and discussion with another author (L.B.) (Figure 1).

### 2.5. Data Extraction

The authors jointly developed a data extraction form to determine which variables to extract. Three authors (G.P., C.M., N.S.) independently extracted the data, discussed the results, and continuously updated the data extraction form in an iterative process. The following data were extracted using a custom-made Excel file: general study characteristics (authors and year), test performed (type of test and timeline), type of lubricant (whether modeling liquid, bonding agent, or others) and mode of application, and type of composite material.

## 3. Results

The information retrieved from the review process is summarized in Table 3.

A total of 295 titles were first extracted from the search of the five databases. After duplicate removal (*n* = 110), additional 167 studies were excluded after reviewing the titles and abstracts as they did not meet the inclusion criteria. The full texts of the remaining 18 studies were examined in detail to determine whether they fulfilled the defined criteria for final inclusion. After full-text reading, two studies were excluded. One paper was retrieved after a further search on reference lists of the full-text reading. Finally, 17 studies were found to be qualified for inclusion in this scoping review (Figure 1).

The following variables were assessed for every paper: performed tests, specimen dimension, application mode of the lubricant, time elapsed before testing, presence of control group, and type of RBCs and lubricants. The variables were recorded in Excel sheets. Missing or not-retrievable variables were entered as “n.r.: not reported”.

Seventeen different Bas were found to be used as a lubricant during restorative procedures, with the 3-step etch-and-rinse (ER) Adper Scotchbond Multi-Purpose Adhesive (3M ESPE, St. Paul, MI, USA) being the adhesive most tested (*n* = 9) [10,13,14,19,20,21,22,23,24].

Eight papers used seven modeling liquids for restorative purposes, with Composite Wetting Resin (Ultradent, South Jordan, UT, USA), Modeling Liquid (GC Corp., Tokyo, Japan), and Bisco Modeling Resin (Bisco, Schaumburg, IL, USA) being the ones most investigated [14,15,16,19,25,26,27,28].

According to the tests performed, lubricants were applied to three different modalities: (1) between two increments for tensile studies (*n* = 4) [14,19,25,29]; (2) between three to four layers (*n* = 6) for color stability analysis, monomer elution, and water uptake studies [10,13,21,22,28,30]; (3) on the top of the outer composite layer (*n* = 7) to evaluate surface hardness, roughness, color stability, and degree of conversion [15,16,20,23,24,26,28]. Five out of the seventeen papers focused on bond strength [10,14,19,29,30]. Eight studies analyzed color stability [10,13,15,21,22,24,26,28], four studies analyzed translucency [10,13,22,24], four papers analyzed micro-hardness [15,16,26,28], three studies analyzed surface roughness [15,26,28], three studies analyzed the degree of conversion [20,23,24], and two studies analyzed flexural strength [10,26]. Isopropyl alcohol (*n* = 3) and ethanol (*n* = 3) were also investigated (*n* = 5) in comparison with Bas and/or MLs [14,20,25,29,30].

The shape of the specimens was dependent on the test performed. The disc was the most used shape to test color stability [10,13,21,22,27,30], monomer elution [10,27,30], roughness [15,26,28], micro-hardness [15,16,26,28], and water uptake [10,30]. Other shapes used for testing were rectangular solid shapes [10,16] and cones [19].

All retrieved studies included a control group, namely, a group in which no lubricant was used.

Thirteen papers waited for 24 h to complete composite conversion [10,14,15,16,19,20,23,24,25,26,27,28,29], while four studies performed tests immediately after the specimen’s preparation [13,21,22,30].

## 4. Discussion

In the present scoping review, in vitro studies evaluating the effects of different lubricants (Bas and MLs) on RBCs’ surface properties were identified. According to the results, the mechanical, optical, and surface characteristics of resin composite materials were evaluated. In general, a shortage of high-quality evidence research was found.

### 4.1. Bond Strength—Tensile Tests

Instruments or brushes wet with lubricants have the intrinsic risk of leaving residues during RBC incremental layering. The influence of lubricants on the cohesive strength of the layer composite has been debated [29]. In the studies analyzed, the lubricants were applied between two composite increments before a tensile test [14,19,25,29]. Tensile tests between layers are mandatory to understand if the use of MLs or Bas affects the overall mechanical performance of the restorative material. Barcellos et al. reported significantly higher bond strength for specimens treated with 3-step ER resinous (non-solvated) monomers (Scotchbond Multi-Purpose, 3M ESPE) [19] with respect to other MLs (Composite Wetting Resin, Ultradent Products; South Jordan, UT, USA; C&B Liquid, Heraeus Kulzer; Hanau, Germany; Adper Single Bond, 3M ESPE; Seefeld, Germany; Prime & Bond NT, Dentsply De Trey; Konstanz, Germany) and the control group (no ML applied between layers). According to the authors, a softening effect of the composite structure and a release of powder particles on the surface were observed after modeling the composite material with the solvated BA (in particular, 2-step ER adhesives), resulting in reduced bond strength values with respect to specimens treated with the above-mentioned solvated BA (Scotchbond Multi-Purpose, 3M ESPE).

Münchow et al. also reported increased cohesive strength for the investigated BA with respect to the control group [10]. In contrast with previous studies, other than 3-step ER non-solvated adhesives, significantly higher bond strength values were reported for liquids containing solvents. Adper Single Bond 2 Adhesive, a more hydrophilic adhesive (2-step ER), showed significantly higher bond strength than the control group (where no ML was used between composite layers). A similar study found higher bonding performances of all the investigated lubricants with respect to the control group, but significantly higher values were reported for solvent-free modeling resin, 3-step ER, and 2-step ER [14]. Conversely, Patel et al. reported that specimens without lubricants (ethanol; 3-step, 2-step, and 1-step Bas) showed statistically higher bond strength. Among lubricants, higher values were reported for 3-step adhesives [30]. Münchow et al. ascribed the increase in tensile strength to the fact that MLs may avoid the occurrence of voids and defects during the layering of the composite [10]. Differences in study design among papers investigating tensile strength may justify the different and conflicting outcomes.

### 4.2. Monomer Elution, Water Sorption

Many papers have investigated the cytotoxicity reactions and biocompatibility of RBCs due to monomer elution [31,32,33]. The monomers and additive components have shown estrogenic, teratogenic, mutagenic, and genotoxic effects [34,35,36]. In addition, the elution of monomers can cause allergic reactions, asthma, allergies, and contact dermatitis [37,38]. The effect of monomer elution from different adhesive systems used as modeling liquids during RBC restorations has also been investigated. Specifically, the adhesives were used between the incremental layering of RBCs and were: one 2-step ER (Adper Single Bond 2, 3M ESPE), one 2-step SE (Clearfil SE Bond, Kuraray Noritake Dental Inc., Okayama, Japan), and one UDMA- and TEGDMA-based ML (composite wetting resin, Ultradent, South Jordan, UT, USA). According to that study, the 2-step SE adhesive and the ML presented significantly higher monomer elution (i.e., UDMA and TEGDMA) with respect to the 2-step ER adhesive. TEGDMA has mutagenic potential and causes chromosomal damage [39], and it is also responsible for exacerbating cariogenic microorganisms’ proliferation [40,41,42]. UDMA does not have carcinogenic and mutagenic effects but can be decomposed into components such as HEMA [43,44], which is carcinogenic and genotoxic [45]. Moreover, a relationship between the amount of released TEGDMA and the degree of conversion of RBCs has also been observed [46].

Among the peculiar properties of composite materials, it is important to consider the capacity of water sorption. These materials are composed of both hydrophilic and hydrophobic monomers, resulting in a certain degree of water sorption. When the water uptake is excessive, phenomena of hygroscopic expansion can lead to deleterious effects on the mechanical and physical properties of the composite itself, such as the swallowing of the resinous matrix, disaggregation at the matrix/filler content interface, and deterioration and the consequent diminished retentive forces. In this regard, Münchow et al. reported a significant reduction of water sorption when a non-solvated adhesive ER 3-step adhesive (Scotchbond Multi-Purpose, 3M ESPE) was used with respect to control (no lubricant) and 2-step ER (Adper Single Bond 2, 3M ESPE) [10]. Conversely, Patel et al. reported significantly higher water uptake for all the investigated Bas (3-step and 2-step ER, 1-step SE) with respect to the control group [30]. The differences between the two studies can be ascribed to the different types of BA and to the different types of specimen treatment before the water uptake procedure.

### 4.3. Optical Properties

#### Color Stability

The optical properties are important parameters to be considered for esthetic composite restorations. Color changes can occur due to water uptake and pigment absorption of staining beverages, aging, or as a result of the interaction between materials [47]. Other than mechanical properties, lubricants may alter the optical properties of RBCs. Indeed, color variations may be caused due to the direct yellowish effects caused by the application of lubricants (irrespective if they are Bas or MLs) or as a consequence of changes in the composition of RBCs after lubricant application.

The results of this review on this issue revealed several papers dealing with the color stability of RBCs used in combination with Bas. No significant color changes were identified by Pereira et al. between specimens treated with an ML (Modeling Resin, Bisco, Schaumburg, IL, USA) or without (control) after submitting the specimens to red wine staining (5 min for 7 days) and brushing (simulating 1 year of clinical service) [28]. Moreover, Araujo et al. found that RBCs modeled with solvated universal adhesive (Adper Universal, 3M ESPE) demonstrated more color stability after artificial staining (thermocycling between grape juice at 58 °C, water at 37 °C, and coffee at 55 °C) compared to a non-solvated hydrophobic one (3-step ER; Adper Scotchbond Multipurpose) [13]. The different viscosity between the two materials investigated has been supposed to influence the formation of defects and void, thus enhancing the mechanical and optical properties of the composite tested [13]. Kutuk et al. reported that an ML caused higher color stability than other investigated liquids (2-step SE primer and UA) after a coffee artificial-staining procedure [15]. Münchow et al. analyzed the color stability of a nano-filled RBC (Filtek Z350 XT, 3M ESPE) submitted to 180 days of artificial coffee staining [10]. Specimens were obtained by layering four increments (0.5 mm), wetting each layer with the lubricant (3-step and 2-step ER) (Scotchbond Multi-Purpose, 3M ESPE, Adper Single Bond 2, 3M ESPE, respectively). The results of their study suggested that the use of an adhesive as a modeling agent may influence the color stability of the composite restoration, irrespective of whether it is stored in water or artificially aged. Control specimens kept color differences within acceptability thresholds after 180 days of water storage. Conversely, ER 2-step group exceeded and the ER 3-step group reached the acceptability threshold (ΔE = 3.3) after 180 days in water storage. Red wine storage showed that the non-solvated adhesive (ER 3-step) showed significantly higher color stability than the control group or the 2-step ER. This finding was also supported by Sedrez-Porto et al., who confirmed significant color stability in specimens (Filtek Z350 XT, 3M ESPE) layered with a solvated adhesive (ER 3-step) (Scotchbond Multi-Purpose, 3M ESPE) between layers with respect to the control group, where no lubricant was used. The same findings but with higher color differences were reported for not-polished specimens, suggesting a possible influence of finishing and polishing procedures on the final shade of the restoration [21]. In another paper, Sedrez-Porto et al. showed that a non-solvated adhesive (3-step ER) had significantly higher color stability than the control group or 2-step ER after 12 months of wine storage [22]. Tuncer et al. reported color differences within the clinical acceptability threshold for specimens treated with an ML on the surface and submitted to 10,000 thermocycles. Melo et al. [24] investigated the effect of two Bas (Adper Single Bond 2 and Adper Scotchbond Multi-Purpose) on the color stability of three RBCs (Filtek Z350XT, IPS Empress Direct, Esthet X HD) after 30 days of water storage. Adper Scotchbond Multi-purpose applied on A2 shade specimens showed less color change in Filtek and Empress Direct with respect to the control group. Conversely, the same adhesive showed higher color change than the control group when applied to Esthet X HD. Both Bas showed an increased color change when applied to bleached shades.

### 4.4. Translucency

Successful esthetic restorations depend on a correct shade selection as well as on the capability to reproduce teeth’s inherent optical characteristics. Among these, translucency plays a fundamental role as it determines the way light interacts with the material itself and with the shade of the underlying substrate. In the current review, the influence of lubricants on opacity/translucency is investigated.

Araujo et al. reported increased opacity stability when a UA was used as an ML for every increment in specimens submitted to thermocycling and staining beverage (grape juice and coffee) storage [13].

Sedrez-Porto et al. reported higher translucency parameter (TP) values at baseline for specimens manufactured with solvated and non-solvated adhesives between layers. In addition, the authors reported higher TP values when a solvated adhesive (ER 2-step) was used as an ML [22].

Similar findings were reported by Munchow et al.: TP values were higher at baseline when solvated and non-solvated adhesives (Scotchbond Multi-Purpose, 3M ESPE; Adper Single Bond 2, 3M ESPE) were used for layered specimens. After 180 days in wine storage, non-solvated adhesives (3-step Ers) showed similar values to the control group, while 2-step Ers showed higher values [10]. Melo et al. reported conflicting results: Esthet X HD showed increased translucency, while Empress Direct showed decreased values when specimens were treated with lubricants (Adper Single Bond 2, Adper Scotchbond Multi-Purpose bonding agent) [24].

### 4.5. Surface Hardness

Bayraktar et al. reported significant micro-hardness reduction when MLs (modeling liquid, composite primer, and modeling resin) were applied (Vickers diamond indenter 0.098 N load for 15 s) on the surface of six resin-based composite specimens, polished before testing. The authors have attributed this result to the diffusion of the modeling resin in the deeper layers, thus influencing hardness values despite the polishing procedures [16]. Micro-hardness tests were also performed by De Paula et al., who reported micro-hardness reduction for all investigated composites (Filtek Z350 XT, 3M ESPE; Empress Direct, Ivoclar, Schaan, Liechtenstein) and lubricants (Scotchbond Multi-Purpose, 3M ESPE, Adper Single Bond 2, 3M ESPE). Nevertheless, in their study, no finishing and polishing procedures were performed. A significantly higher decrease in micro-hardness values was reported for a non-solvated adhesive (3-step ER) [20]. Reduction in hardness may, therefore, be ascribed to the presence of a resin-rich layer that is generally removed with finishing and polishing procedures.

Finishing and polishing procedures have, therefore, a crucial influence on micro-hardness. This finding was also supported by Tuncer et al., who compared the effect of modeling resin with or without surface polishing. For specimens treated with MLs, statistically lower hardness values (Vickers diamond indenter—200 g load for 15 s) were reported for the non-polished specimens. In polished ones, in five out of seven composites, values were comparable to the control group [17]. This finding was also supported by Kutuk et al., who reported no significant differences in micro-hardness (Vickers diamond indenter—200 g load for 10 s) between an ML, a UA, and the control group. Differences were observed when the SE primer (the first liquid of the 2-step SE) was used as a lubricant [15].

In an in vitro study, lubricants showed to be able to preserve micro-hardness (Knoop diamond indenter—50 g load for 15 s) after immersion in staining beverages and brushing simulation procedures [28].

### 4.6. Surface Roughness

Modeling lubricants also have the scope to render the composite surface smooth and shiny. However, only a few papers have investigated the influence of lubricants on RBC surface roughness. Tuncer et al. reported that after finishing and polishing, four out of seven investigated RBCs treated with an ML on the surface reached similar roughness values when compared to the control groups (no lubricant used) [26]. Kutuk et al. reported that ML (Modeling Liquid, GC Corp., Tokyo, Japan) application preserved specimens’ surface roughness values better than other investigated liquids (UA, 2-step SE primer) (G-Premio Bond, GC Corp.; Optibond XTR, Kavo Kerr, OR, CA, USA) after six weeks of coffee storage [15]. Pereira et al. reported significantly higher roughness values for specimens submitted to brushing simulation, regardless of whether specimens were treated or not with an ML [28]. The authors reported that when an ML was used, gloss values were kept higher after staining and brushing simulation with respect to control groups.

### 4.7. Degree of Conversion (DC)

RBCs’ degree of conversion depends on the material’s composition [48]. Whether the lubricants could alter the composition and, therefore, the DC has yet to be investigated in scientific literature. De Paula et al. reported no influence on a nano-filled composite (Filtek Z350 XT, 3M ESPE) and a decreased DC on a nano-hybrid one (Empress Direct, Ivoclar) for all the investigated lubricants (Scotchbond Multi-Purpose, 3M ESPE, Adper Single Bond 2, 3M ESPE) [20]. These findings were partially confirmed by Melo et al. The authors reported lower DC on Filtek Z350 XT (3M ESPE) and on IPS Empress Direct (Ivoclar) with the same BA [24]. Conversely, Dos Santos et al. reported different results [23]: when the same BA of the two above-mentioned papers was used, an increase in DC on IPS Empress Direct was observed.

## 5. Conclusions

Although the use of lubricants is common in clinical practice, information about the effects on RBCs is still scarce. Since the studies revised the lack of standardization of the methodology, the results should be taken with caution. Within the limits of this investigation, the following conclusions can be drawn:

- The use of non-solvated adhesives (3-step ERs) or modeling resins enhances the cohesive bond strength between RBC layers;
- Higher color stability is obtained with non-solvated adhesives or modeling resins between RBC layers;
- Higher RBC translucency values are reported when lubricants are used;
- After lubricant application, finishing and polishing procedures are not always able to provide similar surface properties to specimens treated with no lubricants;
- The effect on the DC depends on the type of lubricant and RBC.
- ML and SE adhesives present higher monomer elution with respect to ER adhesives, although more evidence is needed.
- Whether MLs may increase or reduce water sorption is still controversial.

## Figures and Tables

**Table 1:** Derivative sequence of keywords and MeSH terms used for the search strategy.

Resin-Based Composites	Lubricant
“composite resins” [MeSH]	“wetting agent” [Title/Abstract]
“composite resin” [Title/Abstract]	“lubricant” [Title/Abstract]
“composite resins” [Title/Abstract]	“modeling liquid” [Title/Abstract]
“resin composite “ [Title/Abstract]	“modeling resin” [Title/Abstract]
“resin composites” [Title/Abstract]	“modeler liquid” [Title/Abstract]
	“modeler resin” [Title/Abstract])

**Table 2:** Search strategy as conducted per each database.

Database	Search Strategy
Cochrane	(Composite Resins[MeSH] OR “resin composite*” OR “composite resin*”) AND (“wetting agent” OR “lubricant” OR “modeling liquid” OR “modeling resin” OR “modeler liquid” OR “modeler resin”)
Embase	(‘resin composite*’:ab,ti OR ‘composite resin*’) AND (‘wetting agent’:ab,ti OR lubricant:ab,ti OR ‘modeling liquid’:ab,ti OR ‘modeling resin’:ab,ti OR ‘modeler liquid’:ab,ti OR ‘modeler resin’:ab,ti)
Medline	((composite resins[MeSH Terms]) OR (“composite resin*”[Title/Abstract]) OR (“resin composite*”[Title/Abstract])) AND (“wetting agent”[Title/Abstract] OR “lubricant”[Title/Abstract] OR “modeling liquid”[Title/Abstract] OR “modeling resin”[Title/Abstract] OR “modeler liquid”[Title/Abstract] OR “modeler resin”[Title/Abstract
Scopus	TITLE-ABS-KEY ((“resin composite*” OR “composite resin*”) AND (“wetting agent” OR “lubricant” OR “modeling liquid” OR “modeling resin” OR “modeler liquid” OR “modeler resin”)) AND SUBJAREA(DENT)
Web of Science	ALL = ((“composite resin*” OR “resin composite*”) AND (“wetting agent” OR “lubricant” OR “modeling liquid” OR “modeling resin” OR “modeler liquid” OR “modeler resin”))

**Table 3:** Information retrieved from the review process [10,13,14,15,16,19,20,21,22,23,24,25,26,27,28,29,30].

Author	Performed Tests	Specimen Dimensions	Lubricant Application	Time Elapsed before Testing	Type of Composites	Modeling Liquid Used	Adhesive Used	Other Investigated Liquids
** Tjan AH, Glancy JF, 1988 **	Tensile	Disk 7 × 2.5 mm	Between 2 layers	24 h	Herculite (Kerr/Sybron, Romulus, MI, USA) Bis-GMA; Heliomolar (Vivadent, Tonawanda, NY, USA) Bis-GMA, UDMA	n.r.	Command Resin (Kerr/Sybron, Romulus, MI, USA), Heliobond (Vivadent, Tonawanda, NY, USA), Bondlite (Kerr/Sybron, Romulus, MI, USA) Phosphate esther dentin bonding agent, Adhesit (Vivadent, Tonawanda, NY, USA) Polyurethane dentinal bonding agent	70% Ethanol, 70% Isopropanol
** Perdigăo J, Gomes G, 2006 **	Tensile	10 × 8 × 8 mm	Between 2 layers	24 h	Filtek Z250 (3M Espe, St. Paul, MN, USA) Bis-GMA, UDMA, Bis-EMA	Tescera Sculpting Resin (Bisco, Schaumburg, IL, USA).	Adper Single Bond Adhesive (3M Espe, St. Paul, MN, USA) E&R 2-step; One-Step Adhesive (Bisco, Schaumburg, IL, USA) E&R 2-step; D/E Bonding Resin (Bisco, Schaumburg, IL, USA) E&R 3-step; Scotchbond Multi-Purpose Adhesive (3M Espe, St. Paul, MN, USA) E&R 3-step	Isopropyl alcohol gauze, 70%, Acetone
** Dunn WJ, Strong TC, 2007 **	Flexural-4 point bending	2 × 2 × 24 mm	Between 2 layers	24 h	Filtek Z250 (3M Espe, St. Paul, MN, USA) Bis-GMA, UDMA, Bis-EMA	Unfilled Resin	n.r.	70% Isopropyl alcohol
** Barcellos DC et al., 2008 **	Tensile	2 cones: base = 4 mm, top (luting) 2 mm diameter	Between 2 layers	24 h	Venus (Heraeus Kulzer; Hanau, Germany) Bis-GMA	Composite Wetting Resin (Ultradent Products; South Jordan, UT, USA); C&B Liquid (Heraeus Kulzer; Hanau Germany);	Scotchbond Multi-Purpose Adhesive (3M Espe, St. Paul, MN, USA) E&R 3-step; Adper Single Bond Adhesive (3M Espe, St. Paul, MN, USA) E&R 2-step; Prime & Bond NT (Kostanz, Germany) E&R 2-step.	
** Tuncer S et al., 2013 **	Micro-hardness, Roughness, Color Stability	Discs 8 × 2 mm	Surface application	24 h	GrandioSO (Voco, Cuxhaven, Germany) Bis-GMA, Bis-EMA, TEGDMA; Gradia Direct Posterior (GC America) UEDMA; Aelite LS Posterior (Bisco, Schaumburg, IL, USA) Bis-EMA, TEGDMA; Filtek Silorane (3M ESPE, St. Paul, MN USA); Aelite All Purpose Body (Bisco, Schaumburg, IL, USA) Bis-EMA, TEGDMA; Filtek Ultimate (3M ESPE, St. Paul, MN, USA) Bis-GMA, UDMA, TEGDMA, Bis-EMA; Clearfil Majesty Esthetic (Kuraray Medical Inc., Tokyo, Japan) Bis-EMA, TEGDMA	Bisco Modeling Resin (Bisco, Schaumburg, IL, USA)		
** Münchow EA et al., 2016 **	Water sorption, Solubility, Microtensile, Flexural Strength, Translucency, Color Stability	Several shapes, according to the test	Lubricant on every layer (*n* = 4)	24 h	Filtek Z350 XT (3M Espe, St. Paul, MN, USA) Bis-GMA, UDMA, TEGDMA, Bis-EMA		Adper Scotchbond Multi-Purpose Adhesive (3M Espe, St. Paul, MN, USA) E&R 3-step; Single Bond 2 (3M Espe, St. Paul, MN, USA) E&R 2-step	
**de Paula FC et al., 2016**	Degree of Conversion, Crosslink Density	Discs 5 × 2 mm	Surface application	24 h	Filtek Z350 XT (3M ESPE, St. Paul, MN, USA) Bis-GMA, UDMA, TEGDMA, Bis-EMA; IPS Empress Direct (Ivoclar Vivadent AG, Schaan, Liechtenstein) UDMA, Bis-GMA		Adper Scotchbond Multi-Purpose Adhesive (3M Espe, St. Paul, MN, USA) E&R 3-step; Single Bond 2 (3M Espe, St. Paul, MN, USA) E&R 2-step	70% ethanol
** Patel et al., 2016 **	Water Uptake, Tensile	Discs 5 × 8 mm	Lubricant on every layer (*n* = 4)	0	Solitaire 2 (Heraeus Kulzer, Frankfurt, Germany), Bis-GMA, UDMA, TEGDMA		Optibond FL Adhesive (Kerr UK Ltd., Peterborough, UK) E&R 3-step; Optibond Solo-Plus (Kerr UK Ltd., Peterborough, UK) E&R 2-step, Optibond All-In-One (Kerr UK Ltd., Peterborough, UK) SE 1-step	Ethanol
** Sedrez-Porto et al., 2016 **	Color Stability	Discs 6 × 2 mm	Lubricant on every layer (*n* = 4)	0	Filtek Z350 XT (3M Espe, St. Paul, MN, USA) Bis-GMA, UDMA, TEGDMA		Scotchbond Multi-Purpose Adhesive (3M Espe, St. Paul, MN, USA) E&R 3-step	
** Sedrez-Porto et al., 2017 **	Translucency, Color Stability	Discs 6 × 2 mm	Lubricant on every layer (*n* = 4)	0	Filtek Z350 XT (3M Espe, St. Paul, MN, USA) Bis-GMA, UDMA, TEGDMA		Scotchbond Multi-Purpose Adhesive (3M Espe, St. Paul, MN, USA) E&R 3-step; Single Bond 2 (3M Espe, St. Paul, MN, USA) E&R 2-step	
** Dos Santos et al., 2018 **	Degree of Conversion	Discs 5 × 2 mm	Surface application	24 h	IPS Empress Direct (Ivoclar Vivadent AG, Schaan, Liechtenstein) UDMA, Bis-GMA		Scotchbond Multi-Purpose Adhesive (3M Espe, St. Paul, MN, USA) E&R 3-step; Single Bond 2 (3M Espe, St. Paul, MN, USA) E&R 2-step	
** Melo et al., 2018 **	Degree of Conversion, Color Stability, Translucency	Discs 5 × 2 mm	Surface application	24 h	IPS Empress Direct (Ivoclar Vivadent AG, Schaan, Liechtenstein) UDMA, Bis-GMA; Filtek Z350 XT (3M Espe, St. Paul, MN, USA) Bis-GMA, UDMA, TEGDMA; Esthet X HD (Dentsply Caulk, Milford, DE, USA) TEGDMA, Urethan mod. Bis-GMA		Scotchbond Multi-Purpose Adhesive (3M Espe, St. Paul, MN, USA) E&R 3-step; Single Bond 2 (3M Espe, St. Paul, MN, USA) E&R 2-step	
** Araujo FS et al., 2018 **	Color Stability, Translucency, Whitening index	Discs 10 × 1.5 mm	Lubricant on every layer (*n* = 3)	0	Filtek Z250 (3M Espe, St. Paul, MN, USA) Bis-GMA, UDMA, Bis-EMA		Adper Scotchbond Multi-Purpose Adhesive (3M Espe, St. Paul, MN, USA) E&R 3-step; Adper Universal (3M Espe, St. Paul, MN, USA) Universal Adhesive	
** Kutuk et al., 2020 **	Micro-hardness, Roughness, Color Stability	Discs 12 × 2 mm	Surface application	24 h	Essentia (GC Corp., Tokyo, Japan) UDMA, BisMEPP Bis-EMA Bis-GMA TEGDMA	Modeling Liquid (GC Corp., Tokyo, Japan)	G-Premio Bond (GC Corp., Tokyo, Japan) Universal Adhesive; OptiBond XTR (KavoKerr, OR, California, USA) SE 2-step	
**Bayraktar et al., 2021**	Micro-hardness	Discs 10 × 2 mm	Surface application	24 h	Charisma Smart (Heraeus Kulzer, Hanau, Germany) Bis-EMA, HEDMA, TEGDMA; Estelite Asteria (Tokuyama Dental, Tokyo, Japan) Bis-GMA, Bis-MPEPP, TEGDMA, UDMA; CeramX-One SphereTEC (Dentsply Sirona, Konstanz, Germany) Bis-EMA, TEGDMA; Admira Fusion (VOCO GmbH, Cuxhaven, Germany) Ormocer; Filtek Ultimate (3M, St. Paul, MN, USA) bis-GMA, UDMA, TEGDMA, Bis-EMA; Clearfil Majesty Es-2 (Kuraray Medical Inc., Tokyo, Japan) Bis-GMA, hydrophobic aromatic DMA, and hydrophobic aliphatic DMA	Modeling Liquid (GC Corp., Tokyo, Japan); Composite Primer (GC Corp., Tokyo, Japan); Modeling Resin (KavoKerr, OR, USA)		
** Maalekipour et al., 2021 **	Monomer Elution	Discs 6 × 2 mm	Lubricant on every layer (*n* = 4)	24 h	Filtek Z350 XT (3M Espe, St. Paul, MN, USA) Bis-GMA, UDMA, TEGDMA	Composite Wetting Resin (Ultradent Products; South Jordan, UT, USA)	Adper Single Bond 2 (3M ESPE, St. Paul, MN, USA) E&R 2-step, Clearfil SE bond (Kuraray Noritake Dental Inc., Okayama, Okayama, Japan) SE 2-step	
** Pereira et al., 2021 **	Micro-hardness, Roughness, Gloss, Color Stability	Discs 8 × 2 mm	Surface application	24 h	Filtek Z250 (3M Espe, St. Paul, MN, USA) Bis-GMA, UDMA, Bis-EMA	Bisco Modeling Resin (Bisco, Schaumburg, IL, USA)		

## Data Availability

Not applicable.

## References

[1] Paolone G., Mazzitelli C., Formiga S., Kaitsas F., Breschi L., Mazzoni A., Tete G., Polizzi E., Gherlone E., Cantatore G. (2021). 1 year impact of COVID-19 pandemic on Italian dental professionals: A cross-sectional survey. Minerva Dent. Oral Sci..

[2] Ferracane J.L. (2011). Resin composite—State of the art. Dent. Mater..

[3] Mazzitelli C., Ionescu A., Josic U., Brambilla E., Breschi L., Mazzoni A. (2022). Microbial contamination of resin composites inside their dispensers: An increased risk of cross-infection?. J. Dent..

[4] Josic U., Maravic T., Mazzitelli C., Radovic I., Jacimovic J., del Bianco F., Florenzano F., Breschi L., Mazzoni A. (2021). Is clinical behavior of composite restorations placed in non-carious cervical lesions influenced by the application mode of universal adhesives? A systematic review and meta-analysis. Dent. Mater..

[5] Perdigão J., Araujo E., Ramos R.Q., Gomes G., Pizzolotto L. (2021). Adhesive dentistry: Current concepts and clinical considerations. J. Esthet. Restor. Dent..

[6] Mazzitelli C., Maravic T., Mancuso E., Josic U., Generali L., Comba A., Mazzoni A., Breschi L. (2022). Influence of the activation mode on long-term bond strength and endogenous enzymatic activity of dual-cure resin cements. Clin. Oral Investig..

[7] Mazzitelli C., Maravic T., Sebold M., Checchi V., Josic U., Breschi L., Mazzoni A. (2020). Effect of shelf-life of a universal adhesive to dentin. Int. J. Adhes. Adhes..

[8] Scolavino S., Paolone G., Orsini G., Devoto W., Putignano A. (2016). The Simultaneous Modeling Technique: Closing gaps in posteriors. Int. J. Esthet. Dent..

[9] Paolone G. (2017). Direct composites in anteriors: A matter of substrate. Int. J. Esthet. Dent..

[10] Münchow E.A., Sedrez-Porto J.A., Piva E., Pereira-Cenci T., Cenci M.S. (2016). Use of dental adhesives as modeler liquid of resin composites. Dent. Mater..

[11] Kanter J., Koski R.E., Gough J.E. (1979). Evaluation of insertion methods for composite resin restorations. J. Prosthet. Dent..

[12] Sneed W.D., Draughn R.A. (1980). Effect of alcohol on the strength of a composite resin. Oper. Dent..

[13] Araújo F.S., Barros M.C.R., Santana M.L.C., Oliveira L.S.D.J., Silva P.F.D., Lima G.D.S., Faria-E-Silva A.L. (2018). Effects of adhesive used as modeling liquid on the stability of the color and opacity of composites. J. Esthet. Restor. Dent..

[14] Perdigăo J., Gomes G. (2006). Effect of instrument lubricant on the cohesive strength of a hybrid resin composite. Quintessence Int..

[15] Kutuk Z.B., Erden E., Aksahin D.L., Durak Z.E., Dulda A.C. (2020). Influence of modeling agents on the surface properties of an esthetic nano-hybrid composite. Restor. Dent. Endod..

[16] Bayraktar E.T., Atali P.Y., Korkut B., Kesimli E.G., Tarcin B., Turkmen C. (2021). Effect of Modeling Resins on Microhardness of Resin Composites. Eur. J. Dent..

[17] Ellakwa A., Cho N., Lee I.B. (2007). The effect of resin matrix composition on the polymerization shrinkage and rheological properties of experimental dental composites. Dent. Mater..

[18] Munn Z., Peters M.D.J., Stern C., Tufanaru C., McArthur A., Aromataris E. (2018). Systematic review or scoping review? Guidance for authors when choosing between a systematic or scoping review approach. BMC Med. Res. Methodol..

[19] Barcellos D.C., Pucci C.R., Torres C.R.G., Goto E.H., Inocencio A.C. (2008). Effects of resinous monomers used in restorative dental modeling on the cohesive strength of composite resin. J. Adhes. Dent..

[20] Paula F.C., Valentin R.D.S., Borges B.C.D., Medeiros M.C.D.S., Oliveira R.F., Silva A.O. (2016). Effect of Instrument Lubricants on the Surface Degree of Conversion and Crosslinking Density of Nanocomposites. J. Esthet. Restor. Dent..

[21] Sedrez-Porto J.A., Münchow E.A., Brondani L.P., Cenci M., Pereira-Cenci T. (2016). Effects of modeling liquid/resin and polishing on the color change of resin composite. Braz. Oral Res..

[22] Sedrez-Porto J.A., Munchow E., Cenci M., Pereira-Cenci T. (2017). Translucency and color stability of resin composite and dental adhesives as modeling liquids—A one-year evaluation. Braz. Oral Res..

[23] Dos Santos T.J.S., Melo A.M.D.S., Tertulino M.D., Borges B.C.D., Da Silva A.O., Medeiros M.C.D.S. (2018). Interaction between photoactivators and adhesive systems used as modeling liquid on the degree of conversion of a composite for bleached teeth. Braz. Dent. Sci..

[24] Melo A.M.D.S., Dos Santos T.J.S., Tertulino M.D., Medeiros M.C.D.S., Da Silva A.O., Borges B. (2018). Degree of Conversion, Translucency and Intrinsic Color Stability of Composites During Surface Modeling with Lubricants. Braz. J. Oral Sci..

[25] Dunn W.J., Strong T.C. (2007). Effect of alcohol and unfilled resin in the incremental buildup of resin composite. Quintessence Int..

[26] Tuncer S., Demirci M., Tiryaki M., Ünlü N., Uysal Ö. (2013). The Effect of a Modeling Resin and Thermocycling on the Surface Hardness, Roughness, and Color of Different Resin Composites. J. Esthet. Restor. Dent..

[27] Maalekipour M., Safari M., Barekatain M., Fathi A. (2021). Effect of Adhesive Resin as a Modeling Liquid on Elution of Resin Composite Restorations. Int. J. Dent..

[28] Pereira P., Silva B., Lins R., Lima D., Aguiar F. (2021). Effect of wetting agent coverage on the surface properties of resin composite submitted to brushing and staining cycles. J. Clin. Exp. Dent..

[29] Tjan A.H., Glancy J.F. (1988). Effects of four lubricants used during incremental insertion of two types of visible light-activated composites. J. Prosthet. Dent..

[30] Patel J., Granger C., Parker S., Patel M. (2017). The effect of instrument lubricant on the diametral tensile strength and water uptake of posterior composite restorative material. J. Dent..

[31] Roussou K., Nikolaidis A.K., Ziouti F., Arhakis A., Arapostathis K., Koulaouzidou E.A. (2021). Cytotoxic Evaluation and Determination of Organic and Inorganic Eluates from Restorative Materials. Molecules.

[32] Baldion P.A., Velandia-Romero M.L., Castellanos J.E. (2020). Viability determination data for odontoblast-like cells exposed to resin monomers. Data Brief.

[33] Carrillo-Cotto R., Etges A., Jardim P.S., Torre E., Kaizer M.R., Ferrúa C.P., Nedel F., Cuevas-Suárez C.E., Moraes R.R. (2020). Cytotoxicity of contemporary resin-based dental materials in contact with dentin. Eur. J. Oral Sci..

[34] Geurtsen W. (2000). Biocompatibility of Resin-Modified Filling Materials. Crit. Rev. Oral Biol. Med..

[35] Schweikl H., Spagnuolo G., Schmalz G. (2006). Genetic and Cellular Toxicology of Dental Resin Monomers. J. Dent. Res..

[36] Schwengberg S., Bohlen H., Kleinsasser N., Kehe K., Seiss M., Walther U., Hickel R., Reichl F. (2005). In vitro embryotoxicity assessment with dental restorative materials. J. Dent..

[37] Lindstrom M., Alanko K., Keskinen H., Kanerva L. (2002). Dentist’s occupational asthma, rhinoconjunctivitis, and allergic contact dermatitis from methacrylates. Allergy.

[38] Drucker A., Pratt M.D. (2011). Acrylate Contact Allergy: Patient Characteristics and Evaluation of Screening Allergens. Dermatitis.

[39] Schweikl H., Schmalz G., Weinmann W. (2002). Mutagenic activity of structurally related oxiranes and siloranes in Salmonella typhimurium. Mutat. Res. Toxicol. Environ. Mutagen..

[40] Geurtsen W., Lehmann F., Spahl W., Leyhausen G. (1998). Cytotoxicity of 35 dental resin composite monomers/additives in permanent 3T3 and three human primary fibroblast cultures. J. Biomed. Mater. Res..

[41] Cazzaniga G., Ottobelli M., Ionescu A., Paolone G., Gherlone E., Ferracane J.L., Brambilla E. (2017). In vitro biofilm formation on resin-based composites after different finishing and polishing procedures. J. Dent..

[42] Ionescu A.C., Cazzaniga G., Ottobelli M., Ferracane J.L., Paolone G., Brambilla E. (2018). In vitro biofilm formation on resin-based composites cured under different surface conditions. J. Dent..

[43] Michelsen V.B., Moe G., Skålevik R., Jensen E., Lygre H. (2007). Quantification of organic eluates from polymerized resin-based dental restorative materials by use of GC/MS. J. Chromatogr. B.

[44] Spahl W., Budzikiewicz H., Geurtsen W. (1998). Determination of leachable components from four commercial dental composites by gas and liquid chromatography/mass spectrometry. J. Dent..

[45] Gallorini M., Cataldi A., Di Giacomo V. (2014). HEMA-induced cytotoxicity: Oxidative stress, genotoxicity and apoptosis. Int. Endod. J..

[46] Ferracane J. (1994). Elution of leachable components from composites. J. Oral Rehabil..

[47] Paolone G., Formiga S., De Palma F., Abbruzzese L., Chirico L., Scolavino S., Goracci C., Cantatore G., Vichi A. (2022). Color stability of resin-based composites: Staining procedures with liquids—A narrative review. J. Esthet. Restor. Dent..

[48] Schneider L.F.J., Ribeiro R.B., Liberato W.F., Salgado V.E., Moraes R.R., Cavalcante L.M. (2020). Curing potential and color stability of different resin-based luting materials. Dent. Mater..

